# Strengthening System and Implementation Research Capacity for Child Mental Health and Family Well-being in Sub-Saharan Africa

**DOI:** 10.1007/s40609-021-00204-9

**Published:** 2021-02-02

**Authors:** Anne Mbwayo, Manasi Kumar, Muthoni Mathai, Teresia Mutavi, Jane Nungari, Rosemary Gathara, Mary McKay, Fred Ssewamala, Kimberly Hoagwood, Inge Petersen, Arvin Bhana, Keng-Yen Huang

**Affiliations:** 1 University of Nairobi, Department of Psychiatry, Nairobi, Kenya; 2 BasicNeedsBasicRights, Nairobi, Kenya; 3 Washington University in St. Louis, St. Louis, USA; 4 NYU School of Medicine, New York, USA; 5 University of KwaZulu-Natal, Durban, South Africa; 6 South African Medical Research Council, Cape Town, South Africa

**Keywords:** family strengthening approaches, capacity building, mental health system strengthening, school mental health, implementation science, Kenya

## Abstract

**Background:**

Children in Sub-Saharan Africa (SSA) experience high rates of mental health problems, and the region has limited access to mental health resources and research capacity to address the needs. Despite the success of numerous evidence-based interventions (EBIs) and emerging methodology from the field of implementation science for addressing child mental health needs, most EBIs and implementation science methodology have not been applied in SSA contexts. The SMART-Africa Center aims to address these child welfare, mental health, services, and EBI implementation research gaps by establishing a regional trans-disciplinary collaborative center and studying strategies to strengthening mental health system and implementation research capacity. *Our paper describes the overall framework and strategies that SMART-Africa team developed to strengthen capacity in three SSA countries (Ghana, Kenya, and Uganda)* while focusing on its contextualization for the Kenyan school-community mental health settings. Methods to document the progress and impacts are also described.

**Methods:**

The design of the system and research strengthening activities is guided by a SMART-Africa Capacity Building framework. Two areas of capacity are focused. Mental health system capacity focuses on building political wills, leadership, transdisciplinary partnership, and stakeholders’ global competency in evidence child mental health policy, intervention, and service implementation research. Implementation research capacity building focuses on building researchers’ implementation research competency by carrying out an EBI implementation research (using a Hybrid Type II effectiveness-implementation). For illustration purpose, we describe how the system strengthening strategies has been applied in Kenya, and how the mixed methods design applied to assess the value and impacts of the capacity building activities. Feedback data and evaluation data collection using qualitative and quantitative methods for both areas of capacity building are still ongoing. Data will be analyzed and compared across countries in 2020–2021.

**Conclusion:**

Our work has shown some feasibility of applying the theory-guided system strengthening model in improving child mental health service system and research capacity in one of the three SMART-Africa partnering countries. Our mental health landscape and resource mapping in Kenya also illustrated that capacity building in SSA countries involved complex dynamic, history, and some overlap efforts with multiple partnerships, and these are critical to consider in training activity and evaluation design.

## BACKGROUND

### Child Mental Health Burden and Service System in SSA Contexts

The burden of mental, neurological, and substance use (MNS) disorders account for 10–27% of the Global Burden of Disease (GBD 2017 Disease and Injury Incidence and Prevalence Collaborators, 2018; Murray et al., 2013), and half of all cases of mental disorders develop by age of 14 years (WHO, 2014b). Poor child mental health is associated with a variety of other problems among children and youth , including lower educational achievements and increased engagement in risky behaviors, and these problems often persist into adulthood (Erskine et al., 2015; Peters et al., 2016), affect individuals’ social and economic outcomes later in life, and has enormous consequences on SSA countries’ economic development and international resources (Peters et al., 2016; United Nations Population Fund (UNFPA), 2014).

Child mental health burden in SSA could, in part, be attributed to weak mental health policy/legislation, service delivery and social welfare systems, research capacity, and funding support for research. In relation to *policy, legislation and health expenditure*, only 44% of SSA countries have mental health policy or legislation. Most African countries spend less than 3% of government health expenditure on mental health, and most mental health expenditure (average 71%) is consumed by mental hospitals (Huang, Cheng, Gathibandhe, Bauta, & Akena, 2016; WHO, 2018). The funding is much lower than the WHO recommended 15% minimum. (World Health Organization, 2003). With regard to *existing mental health systems and services* because of low investment, most African countries have a limited number of mental health and social welfare workers, with a ratio of less than 1 per 100 000 population for psychiatrists, nurses, or social workers in most countries, compared to between 10 to 60 in developed countries (Kigozi et al., 2010; WHO, 2014a). At a community level, private health care and the educational and social development sectors also only play a limited role in provision of preventive mental health services (Doku, Wusu-Takyi, & Awakame, 2012; Roberts, Mogan, & Asare, 2014). Few NGOs work directly on community mental health and MNS disorders, but a large number provide mental health services as part of other infectious disease and humanitarian programs, such as counseling in HIV programs and trauma-informed care for refugee, violence, or war conflict affected populations (Henry, 2006; Omar et al., 2010; Walsh, Dawson, & Mattingly, 2010). In relation to *research*, child mental health research is limited because inadequate resources and capacity have been developed or allocated for epidemiological, population-based surveillance, or mental health intervention research. There are few trained mental health implementation or health/social welfare service researchers to support evidence-based intervention (EBI) adaptation, implementation, and evaluation research (Huang et al., 2016). The research funding is also limited, and relies mainly on the priority and funders outsider of the SSA (WHO, 2012, 2013).

### Emerging Global Strategies to Address Child Mental Health Challenges

To address mental health services, policy, and research gaps in SSA countries, several fameworks and strategies have been developed. *For mental health system and service development*, WHO has proposed two frameworks. The WHO’s *Comprehensive Mental Health Action Plan* (WHO, 2013) proposes strengthening governance, providing integrated mental health services in communities, implementing strategies for mental health promotion, and strengthening evidence, research, and information systems for mental health. The WHO’s *Optimal Mix of Mental Health Services Pyramid* framework (WHO, 2009) proposes that the majority of mental health service planning should focus on services outside the traditional healthcare delivery system and in communities because of high need and relatively low cost (WHO, 2009, 2013). *To address intervention research gaps*, recent advancement of *implementation science in global health*, and new emerging evidence for *adopting EBIs from developed to developing countries* have also provided additional strategies to tackle the enormous mental health needs in children (Huang et al., 2017) . For example, several reviews strongly indicate that EBIs can be effective for children from different cultural backgrounds if adapted appropriately with new implementation science methodology (Baumann et al., 2015; Gardner, Montgomery, & Knerr, 2015). *To address policy and research leadership gaps*, adult mental health research in LMICs have generated many strategies *for mental health leadership development* (Abdulmalik et al., 2014; Kleintjes, Lund, Flisher, & MHaPP Research Programme Consortium, 2010; Omar et al., 2010; R4D, 2015; UCH Ibadan, 2015) and/or for *mental health service research collaboration* ((AFFIRM, 2013; Breuer et al., 2014; Center for Global Mental Health, 2011; PRIME, 2015). Despite the potential of these system, implementation research, and leadership development strategies, these strategies have not optimally been applied in child mental health intervention and service research in SSA countries.

### SMART-Africa Initiate

Center for Strengthening Mental Health Systems and Research Training in Sub-Saharan Africa (SMART-Africa) is a SSA regional research center funded by the US National Institute of Mental Health (NIMH) in 2016. The Center aims to address social welfare and mental health needs of children in SSA by establishing a regional transdisciplinary collaborative to support system strengthening and implementation research capacity building. Guided by the global system, implementation, and leadership strategies described above and emerging lessons informed by literature and in consultation with our SSA partners, the SMART-African Center has developed a capacity building activity framework to guide the system changes across three SSA countries (Kenya, Ghana, Uganda). The goals of this paper are to introduce the guiding framework and strategies of our approach for strengthening child mental health/welfare system and implementation research. For illustrative purposes, we describe how this framework has been applied in Kenya.

### Kenyan Context

In Kenya, 75% of population are young people (under 30 years of age), with 22% aged 10–19 and 49% under age 18 (KNBS, 2019; UNFPA Kenya Country Office, 2013). Many children have been exposed to different traumatic events, and are facing high rates of violence (27–41%), poverty (47%), early marriage/childbearing (26–35% married and 18% early childbearing), alcohol use (82%), and unemployment (75% in youth) (Francis, Odwe, & Birungi, 2016; Kiburi, Molebatsi, Obondo, & Kuria, 2018; UNICEF, 2012, 2013). Multiple adversities have contributed to high prevalence of child mental disorders (20–33%) in Kenyan children and youth (Magai, Malik, & Koot, 2018; Ongeri et al., 2018; Osok, Kigamwa, Stoep, Huang, & Kumar, 2018). Targeting community and school mental health as platforms for intervention have been recommended for immediate prioritization (Ireri, White, & Mbwayo, 2019; Mbwayo, Mathai, Khasakhala, Kuria, & Vander Stoep, 2019).

In *policy* Kenya has a mental health plan and legislation developed in early 1990s. Kenyan Mental Health Policy, which provides for a framework on interventions for securing mental health systems reforms, was recently launched and offered key priorities from 2015 to 2030. This is in line with the Constitution of Kenya 2010, Vision 2030, the Kenya Health Policy (2014–2030) and the global commitments (MOH Kenya, 2015). The current policy prioritizes the needs for prevention, and to reduce the prevalence and the impact of mental disorder. It recognizes children and adolescents as one of the vulnerable groups that need for targeted mental health interventions (Atwoli, 2013).

*In research*, there is inadequate data and information on the prevalence of mental and behavioral disorders in representative national child population in Kenya. Also, child mental health psychopathology or epidemiological research and implementation research is limited (Mathai et al., 2019) . There are few trained research personnel to support child mental health epidemiology and service research prior to 2015 (Mathai et al., 2019). Among the published studies that conducted prior to the SMART-Africa initiative, most mental health research has focused on adults and child mental health research was focused on adolescents (Mbwayo & Mathai, 2016) .

*In service*, child mental health service workforce and system is lacking. Even though social workers are part of the mental health system, they are few and therefore reliance is on lay volunteers who are not always optimally trained or integrated. Up until recently, psychologists were also not part of the formal work force (MOH Kenya, 2015).

Given the weak mental health system and research capacity, several efforts have been made at the regional and national-level in mental health system strengthening in Kenya since 2010. Although most of the earlier efforts focused on adult mental health, the capacity established through these efforts are important to consider in the SMART-Africa partnership. Here, we highlight four areas of such efforts. **One**, great strides have been made in Kenyan mental health research during the Fogarty International Center’s Medical Education in Partnership Initiative (MEPI) (2010–2015)(Monroe-Wise et al., 2014). Under this MEPI initiative, attention was drawn to infectious diseases, maternal and child health, and behavioral change/ mental health in a significant manner. Several of our Kenyan key investigators participated in this Initiative and have benefited from mental health research methodology training and the efforts in strengthening capacity of mental health researchers (including psychiatrists, clinical psychologists and psychiatric/social workers) (NIH Fogarty International Center, 2019). **Two**, under the WHO Mental Health Action Plan and Mental Health Gap Action Program (mhGAP) Initiatives, which aim to improve mental health outcomes as part of the Sustainable Development Goals (SDGs) (Daar et al., 2014), a number of innovative collaborations occurred globally, especially on the topics related to capacity building of health/mental health specialists and lay and non-specialists. As part of this global workforce development effort, a number of Kenya-UK based collaborations were created to address similar workforce, health systems and financial barriers in mental health integration in Kenya (Jenkins et al., 2010; Jenkins et al., 2013; Kiima & Jenkins, 2010; Othieno et al., 2009; Semrau et al., 2016). Some of our Kenyan key research and governmental partners were also part of these initiatives. **Three**, Kenyan population’s mental health needs have long been recognized, and mental health research has been targeted or integrated in many areas of health research. For example, over the past decade, while many health and behavioral health interventions have been tested in Kenya (e.g., HIV/AIDS patient engagement, maternal depression, infant feeding, reproductive health, respectful maternity), many researchers recognized the importance of *integrating a mental health component* and *engaging multilevel stakeholders* in health research to achieve greater public health impacts (Kamau, Omigbodun, Bella-Awusah, & Adedokun, 2017; Khasakhala, Ndetei, & Mathai, 2013). **Four**, since 2015 (similar funding timeframe as the SMART-Africa), Kenya has been a site of interest for many international development agencies for testing novel mental health policy, leadership, cost-effective and locally relevant interventions. For example, a collaboration between University of Nairobi (UON) and the University of Washington (UW), which builds on a 30-year HIV/AIDS research collaboration, has extended previous HIV research to include maternal and child mental health, gender-based violence, and HIV related substance abuse research. A number of our Kenyan collaborators are part of this collaboration, and their partnership has yielded a number of accomplishments that has advanced Kenya’s mental health research (Mathai et al., 2019). Similar efforts have being made in parallel by NIMH funded another U19 PAM-D (Partnership for Mental Health Development in SSA: a collaborative initiative for research and capacity building), which further develops collaboration research around mental health in the SSA region (Gureje et al., 2019).

In sum, a decade of mental health research and capacity building efforts in Kenya highlight the numerous strengths that the Kenyan team bring to SMART-Africa Center. The landscape and resource mapping findings also illustrate how solid mental health research competency/capacity has already been established in Kenya and which can be transferred to child mental health implementation research. The findings also suggest that mental health partnerships and system strengthening are not new to the Kenyan investigation team, and their ability to develop efficient partnerships based on their prior experiences and lessons learned. Finally, child and adolescent mental health presents with greater challenges than adult mental health research as it requires involvement of several critical implementing partners like teachers, school, child protection and government, non-governmental and community stakeholders (Mbwayo & Mathai, 2016; Mbwayo, Mathai, Harder, Nicodimos, & Vander Stoep, 2019).

### Goals of this Paper

The goals of this paper are to introduce the guiding framework and strategies of the SMART-Africa approach for strengthening child mental health/welfare system and implementation research. SMART-Africa is aimed to address service gaps and mental health needs of children in SSA by establishing a regional transdisciplinary collaborative to support system strengthening and implementation research capacity building. Given the child mental health system is underdeveloped, under resourced and limited implementation research has been carried out in Kenya on child mental health, our research offers an opportunity to study the feasibility and impact of the SMART-Africa approach in strengthening SSA countries’ child mental health system and implementation research capacity. In this paper, we describe how SMART-Africa framework has been applied in Kenya. We also describe how the mixed methods design is applied to assess the value and impacts of the capacity building activities.

## METHODS

### SMART-Africa’s Approaches for Strengthening Child Mental Health Capacity

#### Guiding Frameworks and Strategies.

**Fig. 1:**
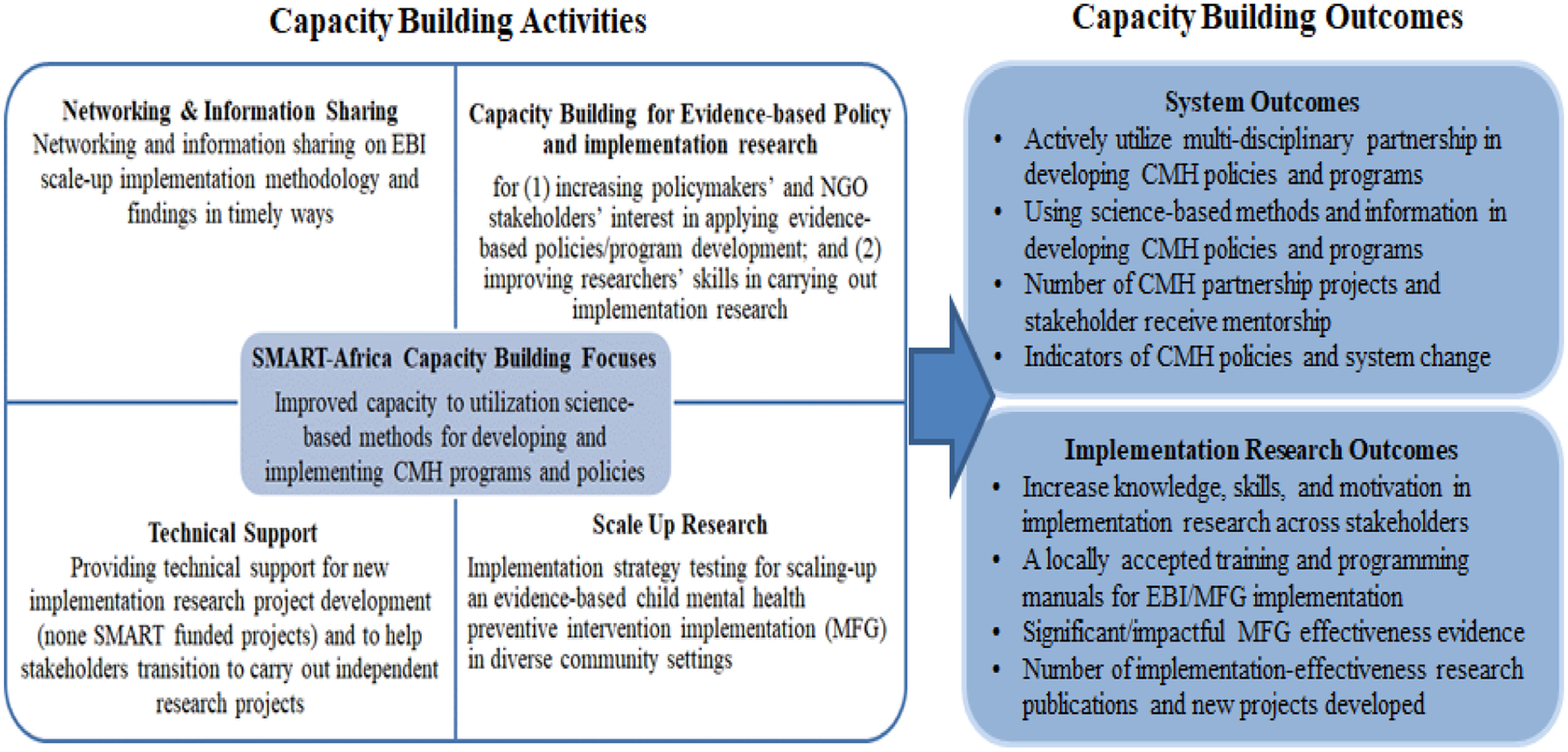
Capacity Building Focuses in SMART-Africa and Hypothesized Impacts

SMART-Africa aims to promote child mental health and address existing system gaps in SSA countries by developing and studying strategies to strengthen mental health system and implementation research capacity. In line with the WHO recommended frameworks and implementation science training methodology described above, SMART-Africa Administrative Core (from the US) and Capacity Building Leads (from South Africa) developed ***a capacity building framework*** (Figure 1) that would simultaneously strengthen two areas of capacity—child mental health system and implementation research capacity in three SSA countries (Ghana, Uganda, Kenya). As shown in Figure 1, the framework includes two targeted areas of capacity building outcomes (system and research outcomes) and four categories of activities (activities for promoting evidence-based policy and implementation research, scale-up research, technical support, and networking/information sharing) that have suggested in the literature and by WHO as effective strategies to strengthen mental health capacity in SSA.

#### Service System and Research Strengthening Activities.

To further map-out activities for two areas of capacity building (system and implementation research), the SMART-Africa US and South Africa leadership team designed a list of activities that are relevant to 4 categories of activities listed in Figure 1. Table 1 detailed the Capacity Building design and activities that are considered for strengthening mental health system and implementation research capacity.

In *mental health system capacity building*, five activities centered on building political will, leadership, trans-discipline partnership teams, and general competency in evidence-based policy, intervention, and service implementation research are focused. Activity design for this area was driven by WHO recommended Comprehensive Mental Health Action and Service Pyramid frameworks (WHO, 2009, 2013) and lessons learned from health system strengthening and leadership development program from international projects (Abdulmalik et al., 2014; Center for Global Mental Health, 2011; Koon et al., 2019; Omar et al., 2010; WHO, 2009, 2013), and effective partnership frameworks (Masseli, Lys, & Schmid, 2005). *implementation research capacity building*, eight activities centered on strengthening child mental health implementation research capacity and generating new EBI effectiveness-implementation evidence are considered. Hands-on experience in carrying out an EBI (Multiple Family Groups/MFG) implementation project in three country sites is structured. As per our design, the activities listed target all aspects of child mental health system barriers listed above, but actual formats vary by countries.

#### MFG.

We chose to adapt MFG for use in SSA because: 1) the EBI addresses one of the most common child mental health problems, disruptive behavioral disorders (DBDs), in the SSA region. Estimated prevalence of DBD in SSA and our study countries ranging from 12 to 33% (Apkan, N.C., & E., 2010; Ashenafi, Kebede, Desta, & Alem, 2001; Cortina, Sodha, Fazel, & Ramchandani, 2012; Huang, Bornheimer, Dankyi, & de-Graft Aikins, 2018; Magai et al., 2018; Nakigudde, Bauta, Wolf, & Huang, 2016); 2) it has been tested with children from families experiencing high adversity, stress, and poverty in the US, African, and HIV/AIDS affected community contexts (Bhana, McKay, Mellins, Petersen, & Bell, 2010; Bhana et al., 2014; Gopalan et al., 2018); and 3) the EBI has shown to be effective in promoting parenting (i.e., family organization, connectedness, support, communication, and discipline practice), improving quality of family life, and reducing child behavioral health challenges (Chacko et al., 2015; Gopalan et al., 2018; Gopalan et al., 2014). Briefly, **MFG** is a community driven and group-based evidence-based psychoeducational and support program that strengthens families to address child disruptive behavior problems or behavioral challenges. MFG utilizes a flexible implementation approach, which can be delivered by parent peers, community health workers, or social welfare/mental health professionals. It can also be implemented in diverse settings, such as social welfare, community-based organizations, schools. MFG was developed based on a body of evidence regarding the influence of parenting and quality of family life on youth behavioral challenges; and it targets multiple risk factors tied to poverty related stress and parenting. MFG focuses on six core areas of contents (4Rs-2Ss): Rules, Responsibility, Relationships, and Respectful Communication and Stress and Social Support. As families discuss their issues around these areas, children and adult caregivers learn to solve problems together and build each other’s capacities. The approach enables the high-risk problem behaviors to be understood and tackled but also provide some common learning around internalizing and externalizing disorders at home (Bhana et al., 2010; Gopalan et al., 2018).

#### MFG Implementation Research Design.

To strengthen implementation research capacity and generate new effectiveness-implementation evidence in SSA, the same three-arm Hybrid Type II effectiveness-implementation research (Curran, Bauer, Mittman, Pyne, & Stetler, 2012) and Reach-Effectiveness-Adoption- Implementation-Maintenance (RE-AIM) evaluation design (Glasgow, Vogt, & Boles, 1999) are applied to three study countries, and same evaluation tools (with language adaptation) are used for evaluation. Across countries, MFG is tested in school settings using two task-shifting implementation approaches: (i) implemented by community health workers/teams (proposed to be under Ministry of Health structure) and (ii) implemented by school community members (parent-peers or teachers from school communities, to be determined by study countries). The effectiveness findings from these two implementation approaches would be compared with control condition, which receives mental health materials for awareness promotion. Given the differences in research capacities across countries, Uganda would carry out a scale-up research study in 30 schools, and Ghana and Kenya would carry out a small scale (pilot) implementation-effectiveness study in three schools. Detailed descriptions about MFG effectiveness-implementation trial design and evaluation methods/measures can be found in the SMART-Africa Trial article (Ssewamala et al., 2018).

#### Additional Capacity Building Feature Considered.

In addition to the capacity building activities described above, several additional design features were carefully considered. To support the capacity building activities, SMART-Africa brings *rich expertise and resources* from multidisciplinary scholarships from both the USA and African regions (South Africa, Ghana, Uganda, Kenya) to support the training. The team of experts from all participating countries have contributed to a range of capacity building activities such as mentorship, technical support, and offering workshop training. We recognize country level variations and each country’s strengths, but at the same time *recognize local specific needs and local governments’ priority differences* We allow capacity-building activities or stakeholder meeting discussion topics to vary based on the needs and priorities, and provide technical implementation methodology support or knowledge sharing. We also emphasize *strength building, network learning, and SSA local stakeholder collaboration*, which recognize each country’s unique mental health capacity strengths and experiences, and contribution to the network of knowledge. Our network learning approach draws on existing partnerships in the four countries to create child implementation science “laboratories”, which offers the opportunity to experiment both within and across country systems. Furthermore, to create efficient communication and learning opportunities, we utilize *distance learning/communication technologies* as well as group- and individual-based mentorship support and learning opportunities to share implementation science related knowledge and research skills. The *eclectic use of multiple training methods* (i.e., webinars, mentorship support), facilitate knowledge sharing and translation of skills to action across SSA countries and between US and SSA countries (O’Neil et al., 2015).

#### Evaluation.

Table 1 “Methods for Assessing Capacity Building Model” column outlines our indicators of success and methodology applied for evaluation in two capacity building areas. In general, a mixed methods evaluation design is applied. The impact assessment for the System capacity building activities is based on qualitative data (from researchers and stakeholders). For implementation research capacity building outcomes, the primary outcomes will be evaluated based on implementation effectiveness data that apply the same quantitative measures used in the scale-up study site-Uganda (Ssewamala et al., 2018). Additional feedback, satisfaction, partnership quality, and product data will be gathered in Year 5 to capture qualitative experience.

### Applications to Kenya

In Kenya, the in-country team is led by a group of 4 key faculty/researchers from University of Nairobi, and in partnership with representatives of Ministry of Health and Education, and NGOs. In this section, we describe the application of the SMART-Africa system strengthening framework and strategies in the Kenya context. We describe the activities, ways, and procedures that the Kenyan team applies for the capacity building.

#### Procedures in Applying SMART-African System Strengthening Approach.

Given that Kenyan research investigators (from UON) already have substantial mental health epidemiological research competency and partnership experiences from prior health/mental health research capacity building projects, we structured learning opportunities specifically around child mental health, and build on the existing strengths and the Kenyan partnership team’s needs (Kumar & Sensoy Bahar, 2019). Specifically, we identified areas to be focused with Kenyan team first (described next), and mapped out the activities based on Table 1 capacity building activity design framework. Table 2 (the second right column) lists the capacity building activities that have been or will be conducted in Kenya.

Capacity building activities identified to be focused on for Kenyan team include : **i)** developing and managing a new policy-academic-NGO-community multi-stakeholder partnership that engage stakeholders from government (MOE, MOH), research, NGOs (Basic Needs, World Vision), schools (teachers, head teachers), and communities (children and families) for child mental health policy and service research discussion; which would be an extension of prior broader mental health research network/collaboration; **ii)** implementation science methodology learning and applications to child mental health (including frameworks, methods for developing multi-level implementation strategies, methods for EBI content and procedure adaptation/ modification to fit local contexts/resources/needs; implementation-effectiveness evaluation design/frameworks); **iii)** developing an implementation collaboration structure (e.g., multi-team implementation research structure, task-sharing approach of implementation practice-research collaboration between UON and NGO Basic Needs) to effectively conducting and managing multi-components of implementation research projects; **iv)** new child mental health service and implementation research projects development (based on interests expressed by partnering stakeholders and researchers, e.g., around adolescent mental health, mhGap and mHealth in child mental health); **v)** child mental health resource development, testing, and sharing (e.g., mental health awareness materials; screening/assessment tools) that can be used broadly used in diverse Kenya contexts; and **vi)** strategies to strengthening school mental health evidence and service research to support school mental health policy decision and program design.

As described in Table 2, most system capacity building activities were conducted in group-training format and carried out during the SMART-African Annual meetings that usually take-place during July or August and last for 3–4 days. After attending the training and learning new knowledge, the research leadership team would conduct additional activities/meetings in their own country to share knowledge and lessons learned with the in-country partnering stakeholders (with or without the support from the SMART-administrative core). For implementation research capacity building activities (hands-on experience), the Kenyan team was focused on need/implementation context assessment in Year 1, content adaptations and refinement (for community health worker training manual, family group manual; procedure) in Year 2 and 3, research and implementation team training in Year 3, and effectiveness-implementation study in Year 4. As part of the implementation research activities, Kenyan team has developed an adapted version of MFG, which has been named *Familia Pamoja* in Swahili and *Families Together* in English. The MFG implementation and evaluation research is ongoing. Table 2 lists the capacity building activities that have been conducted in Kenya. Most activities were consistent with the activities outlined in the protocol and needs. Table 2 results support some feasibility in applying the SMART-Africa Protocol. The impact evaluation in Year 5 will further assess the feasibility, quality, acceptability, and usability of the SMART-Africa approach.

#### Current Research Status and Implications.

In Table 2 right column, we describe the sources of data that have been collected/ documented by Kenyan research team. We also provide preliminary usability information based on the activities and milestones that have been achieved. SMART-Africa is in its 4^th^ Year of research. The Kenyan team has prioritized a few areas of capacity building activities listed in Table 2 for the remaining funding period.

- *Completing MFG Effectiveness-Implementation Research Activities and Evaluation Study*. The Kenyan team is now focusing on Stage 2 capacity building activities by carrying out a MFG effectiveness-implementation study. We plan to complete the post MFG evaluation data collection, data analysis, and disseminate the findings in 2021.
- *Policy-academic-NGO-community multi-stakeholder partnership and stakeholder engagement*. This paper is the first step towards establishing a SMART-Africa Kenya conceptual and working model. We intend to invite stakeholder participation in refining the system strengthening conceptual model so that different technical working groups of the Ministries of Health, Education and Social Services can benefit from multi-sectoral partnerships on child mental health issues using a model sensitive to Kenyan’s needs.
- *Further strengthening inter-ministerial collaboration on child mental health*. The Kenyan Ministry of Health is in principle meant to provide technical support on mental health including child and adolescent mental health to other relevant Ministries including Education. There are few entry points around mental health and the programming remains regimented towards integrated special needs pupils - those experiencing developmental and neurocognitive disabilities. We plan to work collaboratively with the Ministries to build on each other’s capacities, train in child mental health issues and integrate EBIs like *Familia Pamoja* into their work.
- *Developing psychoeducational materials for later dissemination*. The team has developed psychoeducational materials with a view to strengthening the existing gaps through the use of simplified information sheets in control schools and community settings. These materials were developed by clinical psychology students under guidance from the team and proofread by psychiatrists, senior MOH and MOE program officers and teachers, and materials are ready for broader dissemination in Kenya.
- *Engaging Basic Needs (the partnering NGO) on disseminating Familia Pamoja*. The involvement of Basic Needs on MFG implementation and co-leading the implementation-effectiveness research is a strategic part of capacity building of SMART Kenya and the consortium as such. Basic Needs will also be playing an instrumental role in the future scale up of the MFG model.

In addition to completing the activities, the Kenyan team is planning to gather additional feedback/satisfaction, acceptability, usefulness, partnership quality, and impact data (academic, mental health service, and policy accomplishments/products from the SMART-Africa) from participating stakeholders (researchers, NGOs, governmental partners) in Year 5 to provide overall progress and lessons learned to inform future child and mental health implementation research. There are also a few activities have been identified to continue after the project end.

- *Additional implementation focuses on testing Familia Pamoja*. As this work develops further, the in-country team is also looking to explore and agree on gaps in mental health policy in Kenya and identify points of entry to strengthen Kenyan child mental health policy. In this work, the Kenyan Ministry of Health is very supportive. By utilizing expertise of the consortium, the effort is to produce epidemiological and policy relevant data that can provide pointers to sharpening guidelines and assessments for Kenyan children in school, home and community settings. Another element of this work is to explore and agree on appropriate interventions for Kenya’s school-based mental health system and the team is looking at different ways of bolstering multi-stakeholder foci (Mbwayo, Mathai, Khasakhala, et al., 2019).
- *Developing additional referral resources and pathways for high need families*. The Kenyan mental health collaboration team is considering development of ways of addressing referral pathways when any distressed pupil is identified. A Standard Operating Principles (SOPs) handbook will be specifically developed to provide local and credible referrals.
- *Translating and adapting the manual*. The Kenyan team intends to contextualize the manual and adapt it further by making amendments to Kiswahili, use of culturally specific terms, formatting that is user friendly and activities responsive to parent-child working together in groups.
- *Further developing implementation strategies*. We plan to compile lessons learned from stakeholders and MFG implementers and users, and developing additional implementation strategies for consideration in future MFG or school-based mental health service and implementation research.

## CONCLUSION

This paper describes the applications of the child mental health system and implementation research capacity building framework developed by the SMART-African Center and implemented in the Kenyan contexts. As illustrated in the paper, we show some feasibility of applying this framework in one of the three SMART-Africa partnering countries. In addition, our mental health landscape and resource mapping in Kenya also illustrated that capacity building in SSA countries involved complex dynamic, history, and some overlap efforts with multiple partnerships, and these are critical to consider in training activity and evaluation design. For example, in the landscape/system context analysis, we illustrated that many Kenyan researchers have involved in global research capacity-building efforts and have gained solid mental health research competency/capacity that can be transferred to child mental health implementation research. Overlook these strengths and existing capacity building efforts may hinder the development of effective North-South research partnership. Our paper also illustrates that multiple research capacity-building and collaboration efforts are occurring concurrently in Kenya, which may pose challenges in evaluating impacts of the capacity building efforts. We plan to apply qualitative data collection method to unpack how the dynamic and history influence SMART-Africa US-Kenya partnership, and better understand what the unique contribution that SMART-Africa contributes to child mental health system and implementation research in Kenya.

Although this study did not provide descriptions for applying the framework to other SMART-Africa partnering countries (in Uganda and Ghana), we anticipate the applicability of the framework (Figure 1) and design (Table 1) to these two SSA countries may be similar, especially in ways of structuring/designing activities. Our statement is based on similar child mental health service and research context, and feedback that we learned from cross-site partnership meetings. We plan to discuss our experience with other partnering countries to share lessons learned.

## Figures and Tables

**Table 1.** SMART-Africa Capacity Building Protocol for Strengthening Child Mental Health (CMH) System and Implementation Research

Areas of Capacity Building	Capacity Building Activities in the Protocol	Methods for Evaluating Capacity Building Approaches (Indicators of Success & Evaluation Methods)
**CMH System Capacity Building:** Building political wills, leadership, trans-disciplinary partnership, and stakeholders’ global/general competency in evidence-based CMH policy, intervention, and service implementation research (Primary focus in Year 1 and 2) **Activity design based on WHO recommended and implementation science training frameworks (WHO, 2009, 2013)	**To establish and engage a trans-disciplinary research consortium of government, NGO, community and academic stakeholders in Uganda, Ghana, and Kenya to focus on understanding and making plans to address CMH burden** through the following activities: To establish partnerships and facilitate discussion and knowledge exchange for cross-discipline stakeholders in participatory planning for CMH services. Prioritizing the identified challenges and making plans for resolving service gaps (e.g., design educational/mental health awareness materials) Carrying out plans to reduce CMH research or service gaps (prioritizing policy questions for study, developing assessment system to inform decisions, conduct pilot feasibility research studies)	**Indicators of success** Number of stakeholders participated in workshops/meetings Develop a plan or prioritization for solving CMH gaps **Eval. Methods**: Qualitative data from engagement and stakeholder meetings throughout grant period
	**To increase researchers/trainees/policy/NGO stakeholders’ knowledge and competency in CMH service and implementation research via variety of instructional/learning formats** (i.e., SMART-Africa annual conference, Global Mental Health conference/hub meetings, webinars, web resources, workshops) and gaining hands-on experience in designing and carrying out implementation projects	**Indicators of success** Increase knowledge, skills, and motivation in implementation research across stakeholders Actively utilize multi-disciplinary partnership method for program and project development (number of implementation collaborative projects and funded implementation grant) Actively utilize science-based methods in developing CMH policies and program (number of program applied this approach) Number of partnership CMH project and stakeholder receive mentorship Indicators of CMH policies and system change **Eval. Methods**: Qualitative data/report from partnering stakeholders/fellows. Peer-reviewed-papers, partnership, and grant record tracking data from annual progress report.
	**To increase policy/governmental and NGO stakeholders’ utilization of science-evidence-based methods** for developing CMH policies and programs and adaptation to become informed consumers of research.	
	**To provide technical support** to SSA trans-disciplinary leaderships in developing CMH implementation projects and facilitate their transition to an active users	
	**To provide fellowship and mentorship programs** for a group of highly promising cross-discipline junior and senior faculty, technical personnel, and graduate MPH/PhD students who are committed to pursuing a research career in CMH implementation research.	
**CMH Implementation Research Capacity Building:** To build CMH implementation research capacity for researchers and technical personnel to conduct an EBI/MFGs implementation research. (Primary focus in Year 3, 4 and 5) **Activity design based on implementation phases and CFIR domains (Aarons, Hurlburt, & Horwitz, 2011; CFIR Research Team, 2014; Koon et al., 2019)	**To understand and documenting the multi-level contexts of CMH system and research in study countries (Understand pre-condition)**: Each country team would characterize policy, CMH system, school mental health system, resources, CMH EBI research, target consumers perception about CMH and needs, fit of the EBI/MFG in country context.	**Indicators of success** A locally accepted training and programming manuals for MFGs/the EBI implementation A locally accepted implementation approach and process that fit local school and CMH resources **Eval. Methods**: Qualitative data and quantitative data (feedback/satisfaction/acceptability data) from implementers and users/families
	**To understand the fit and readiness of targeted setting for implementing the selected EBI/MFGs**: Understand implementers’ and target consumers’ (school leaders, teacher, family, community-health-workers/CHWs, NGO implementer) perception about MFGs, resources, and fit for implementing MFGs. The information would be used to inform EBI content and process adaptation.	
	**To adapt the EBI/MFGs**: Working collaboratively with NGO implementation partners and CMH stakeholders in adapting CHW training and family group manuals, evaluation tools, and processes	
	**To recruit s**tudy sites/schools, families, CHWs, and research assistants for implementation and evaluation	**Indicators of success** Increase knowledge, skills, and competency in conducting implementation research in researchers and program implementers Significant/impactful MFG effectiveness evidence Number of implementation-effectiveness research publication and cross-country join publication Number of new CMH implementation study/project and new grant applied **Eval. Methods**: Quantitative evaluation data from MFG evaluation study; qualitative and feedback data from individual and group meetings with implementers, researchers, and families.
	**To training the trainers, facilitators** (CHWs)**, and research assistants for MFGs implementation and evaluation**: First, academic researchers and NGO lead implementers would be trained on MFGs by EBI developers. Upon the completion of the training, NGO lead implementers will provide training to MFG facilitators (two types of CHWs: Village-Health-Teams and Parent-Peers). A team of research assistants would also be trained by country PI/Co-Is on conducting implementation and effectiveness outcome evaluation research.	
	**To carry out implementation-effectiveness evaluation study**: MFGs would be implemented in study countries in Years 3 and 4 using the modified/adapted version of MFGs. Given the variation of resource and implementation capacity across countries, Uganda would carry out a scale-up research study in 30 school sites, and Ghana and Kenya would carry out a small scale implementation study in 3 school sites.	
	**To provide a network of learning opportunities across implementation countries (for feedback, quality improvement, and refinement purposes)**: Monthly meetings across study countries would be utilized for cross-country implementation experience sharing, implementation strategy learning, research findings sharing, and technical solution inputs. Each country team would also meet with the US SMART-African administrative core and NIMH leaders monthly to discuss experience and provide update.	
	**Implementation evidence sharing, service improvement, and future scale-up, and sustainment projects planning**: Conducting regional, national, and global dissemination through multiple communication channels	

Note. CMH=Child mental health; CFIR= consolidated framework for implementation research; MFG= Multiple Family Groups; Eval. = evaluation. Detail description about MFGs and Hybrid Type II design for two CHW implementation approaches can be found in the Ssewamala, et al. article (comparing the program implemented and effectiveness outcomes when the MFGs is implemented by the village health teams vs. parent peer)

**Table 2.** SMART-Kenya Capacity Building Activities, Milestone, and Research Status Update

Areas of Capacity Building	Capacity Building Activities in Protocol	Activities Conducted in Kenya	Usability of Capacity Building Model
**CMH System Capacity Building:.** Building political wills, leadership, trans-disciplinary partnership, and stakeholders’ global/general competency in evidence-based CMH policy, intervention, and service implementation research (Year 1–2)	To establish and engage a trans-disciplinary research consortium of government, NGO, community and academic stakeholders in Uganda, Ghana, and Kenya to focus on understanding and making plans to address CMH burden	Kenyan PI/Co-Is and key NGO/Policy stakeholders attend SMART-Africa Annual Conferences & meetings (3–4 days per year) in Uganda Engage and establish Kenya Child Mental Health multi-disciplinary partnership group in Year 1 (with governmental [MOE/MOH]-NGO-academic stakeholders), and carry out bi-annual stakeholder meetings. Additional need-based meetings also conducted. Introducing SMART-Africa capacity building and partnership models, the intervention implementation design, and the scale-up approaches to stakeholders Collaborate with Kenyan stakeholders to develop SMART Kenya’s goals Explore and agree on gaps in mental health policy in Kenya and identify points of entry to address child mental health policy Government stakeholder shared their perspective about priority (MOH: prevention and promotion efforts, training teachers and parents in MH literacy, local leadership and ownership of CMH programs as the country priority; MOE: training teachers and parents to understand child wellbeing, inclusive education-none discriminating children with disabilities, providing mental health first aid and support at school itself) Explore and agree on appropriate interventions for Kenyan school based mental health systems with stakeholders Define and establish a SMART Kenya conceptual and working model Discuss how to establish a SMART Africa hub resource leveraging the strengths and needs of Kenya as a partner Recognize “We Know a lot, but we need to ACT”. Making commitment in closing the gaps by Listening, Learning, and Implementing (or Listening to learn, Learning to doing; Implementing for sharing/dissemination) and “Strength to Strength”.	**Milestones evaluation** and tracking is ongoing (All activities have been provided, and stakeholders participated most activities). **Eval data**: Qualitative data (meeting/discussion notes; focus groups data) were collected from stakeholders and meetings. Additional qualitative feedback/ satisfaction data will be gathered in Year 5 focused on experiences across years.
	To increase stakeholders’ knowledge and competency in CMH implementation and service research via variety of learning formats	Gaining knowledge by participating SMART-Africa Annual conferences and NIMH Global Mental Health Conference, Global Hub meetings, webinars, and web resources Provide workshops to discuss effective partnership (e.g., partnership types, stages, how to build effective and trusted partnership relationship) Workshop on child/adolescent centered mental health care	**Milestones evaluation** and tracking is ongoing (All activities have been provided, and key stakeholders have participated in most listed 2–5 activities. **Eval data:** Qualitative data (from individuals and focus groups) and meeting record data (meeting/ discussion notes) are collected and available for analysis. Additional qualitative feedback/ satisfaction data will be gathered in Year 5 focused on experiences across years.
	To increase policy and NGO stakeholders’ utilization of evidence-based methods for CMH policy and program development	Provide workshops to policy/government, NGO stakeholders on implementation research; child and adolescent friendly/centered services, and science approach to develop digital health Provide update information on SMART-Africa projects and approaches, and invite policy relevant discussion in stakeholder meetings.	
	**To provide technical support** in developing CMH implementation projects and facilitate to be an active users for evidence-based policy development	Provide technical support to NGOs on grant submission on the topics related to adolescent violence and digital mental health Working collaboratively with NGOs to develop/submit grants or with government stakeholders on new mental health service improvement projects Monthly skype call with the US Leadership team	
	**To provide fellowship and mentorship programs** for highly promising cross-discipline junior researchers	Recruited a PhD level social work fellow (as part of the SMART mentorship program) Provide mentorship support to researchers on NIMH grant writing (e.g., K43, R grant) Provide mentorship opportunities to graduate-level students (by including them in SMART-Kenyan training opportunities, research projects, providing support on child mental health paper writing)	
**CMH Implementation Research Capacity Building: .** To build CMH implementation research capacity for researchers and technical personnel to conduct an EBI/MFGs implementation research (Year 3–4)	To understand and documenting the multi-level contexts of CMH system and research in study countries	Conduct literature review and carry out needs assessment to characterize CMH system, school mental health system, resources, EBI research, and community needs from diverse stakeholders’ perspectives.	**Milestones evaluation** and tracking (All activities have been conducted, and a localized training and MFG program manuals have been developed for testing. **Eval data:** Qualitative data (from stakeholders) and meeting record data (meeting/discussion notes) have been collected and available for analysis
	To understand the fit and readiness of targeted setting for implementing the EBI/MFG	Gather data from school leaders, teacher, family, community-health-workers/CHWs, NGO implementer to understand implementers’ and target consumers’ perception about MFG, resources, and fit for implementing MFG.	
	To adapt the MFG, and develop a Kenyan version of MFG (Familia Pamoja= Families Together)	Work collaboratively with the NGO/Basic Needs implementation partner, teachers, and parents in adapting MFG CHW-training manual and family-group manual to fit Kenyan context The Kenyan PI/Co-Is develop child mental health literacy/awareness materials to promote mental health awareness. The materials were reviewed by MOE/MOH stakeholders, and shared with MOE/MOH for broader dissemination Adapt and translate evaluation tools Adopt implementation and research processes to fit with available resources and structures	
	**To recruit s**tudy sites/schools, families, CHWs, and research assistants for implementation and evaluation: developing recruitment strategies and carry out recruitment according to experimental study design	Obtain the school list from district education office and select schools based on design Recruit 3 primary schools and in collaboration with school staff to develop recruitment strategies and carried our family recruitment. To create three task teams to lead different portion of implementation research efforts: (1) Implementation team recruits CHWs and parent peers from community and schools based on recruitment criteria; (2) Quality monitoring team recruits fidelity observers; (3) research evaluation team recruits research assistants for evaluation data collection	**Milestones evaluation** and tracking is ongoing (MFGs implementation is ongoing; most activities have been accomplished; and stakeholders have participated most listed 2–5 activities. **Eval data:** Qualitative data (from individuals and focus groups) and meeting record data (meeting/ discussion notes) are collected and available for analysis. Additional qualitative feedback/ satisfaction data will be gathered in Year 5 focused on experiences across years.
	**To training the trainers, facilitators** (CHWs)**, and research assistants for MFG implementation and evaluation**:	Obtain the school list from district education office and select schools based on design Using train-the-trainer model, the academic researchers (PI/Co-Is) and NGO implementers receive MFG training from Ugandan EBI implementers/with support from the developers. The trained NGO lead implementers provide training to CHWs (Village-Health-Teams and Parent-Peers), with support from PI and research team. A team of research assistants are trained by the lead Co-Is on conducting implementation and effectiveness outcome evaluation	
	To carry out implementation-effectiveness evaluation study	Formally carry out MFG implementation-effectiveness study in Years 4 using the modified/adapted version of MFGs. Hands-on experience in applying RE-AIM framework in evaluation research	
	To provide a network of learning opportunities across implementation countries (for feedback, quality improvement, and refinement purposes)	Monthly meetings across study countries (lead by SSA countries’ researchers) for cross-country implementation experience sharing, implementation strategy learning, research findings sharing, and technical solution inputs. Country team met with the US SMART-African administrative core and NIMH leaders monthly to share experience, provide update, and discuss challenges & solutions Yearly invitation for collaborators from Kenya and Ghana to visit the Ugandan scale-up study site to learn from Ugandan experience. Continue monthly skype call with the US Leadership team Monthly call cross 3 study countries to share implementation experience and provide technical support from other countries	
	Implementation evidence sharing, service improvement, and future scale-up, and sustainment projects planning:	***Planned activities (not yet happen)*** Carry out implementation-effectiveness evaluation analysis, and apply results for future policy implementation or policy decision Conducting regional, national, and global dissemination through multiple communication channels (such as web, regional and global child mental health conferences)	

***Note.*** CMH=Child mental health; CHW=Community health worker; CFIR= consolidated framework for implementation research; MFG= Multiple Family Groups; Eval. = Evaluation.

## Abbreviations:

SSA: Sub-Saharan Africa
EBI: Evidence-based Interventions
RE-AIM: Reach, Effectiveness, Adoption, Implementation, and Maintenance
MNS: mental, neurological, and substance use
WHO: World Health Organization
MFG: Multiple Family Groups
DBD: Disruptive Behavioral Disorders
